# Immuno-informatics study identifies conserved T cell epitopes in non-structural proteins of Bluetongue virus serotypes: formulation of a computationally optimized next-generation broad-spectrum multi-epitope vaccine

**DOI:** 10.3389/fimmu.2024.1424307

**Published:** 2024-07-01

**Authors:** Harish Babu Kolla, Mansi Dutt, Anuj Kumar, Roopa Hebbandi Nanjunadappa, Tobias Karakach, Karam Pal Singh, David Kelvin, Peter Paul Clement Mertens, Channakeshava Sokke Umeshappa

**Affiliations:** ^1^ Department of Microbiology, Immunology and Pediatrics, Dalhousie University, Halifax, NS, Canada; ^2^ Immunology Division, IWK Health Centre, Halifax, NS, Canada; ^3^ Department of Pharmacology, Dalhousie University, Halifax, NS, Canada; ^4^ Center for Animal Disease Research and Diagnosis, Indian Veterinary Research Institute, Bareilly, India; ^5^ School of Veterinary Medicine and Science, Pirbright Institute-Surrey, Woking, United Kingdom

**Keywords:** Bluetongue virus serotypes, non-structural proteins, conserved CD8+ and CD4+ T cell epitopes, in silico broad-spectrum BTV vaccine formulation, immunoinformatics

## Abstract

**Introduction:**

Bluetongue (BT) poses a significant threat to the livestock industry, affecting various animal species and resulting in substantial economic losses. The existence of numerous BT virus (BTV) serotypes has hindered control efforts, highlighting the need for broad-spectrum vaccines.

**Methodology:**

In this study, we evaluated the conserved amino acid sequences within key non-structural (NS) proteins of BTV and identified numerous highly conserved murine- and bovine-specific MHC class I-restricted (MHC-I) CD8+ and MHC-II-restricted CD4+ epitopes. We then screened these conserved epitopes for antigenicity, allergenicity, toxicity, and solubility. Using these epitopes, we developed in silico-based broad-spectrum multiepitope vaccines with Toll-like receptor (TLR-4) agonists. The predicted proinflammatory cytokine response was assessed in silico using the C-IMMSIM server. Structural modeling and refinement were achieved using Robetta and GalaxyWEB servers. Finally, we assessed the stability of the docking complexes through extensive 100-nanosecond molecular dynamics simulations before considering the vaccines for codon optimization and in silico cloning.

**Results:**

We found many epitopes that meet these criteria within NS1 and NS2 proteins and developed in silico broad-spectrum vaccines. The immune simulation studies revealed that these vaccines induce high levels of IFN-γ and IL-2 in the vaccinated groups. Protein-protein docking analysis demonstrated promising epitopes with strong binding affinities to TLR-4. The docked complexes were stable, with minimal Root Mean Square Deviation and Root Mean Square Fluctuation values. Finally, the in silico-cloned plasmids have high % of GC content with > 0.8 codon adaptation index, suggesting they are suitable for expressing the protein vaccines in prokaryotic system.

**Discussion:**

These next-generation vaccine designs are promising and warrant further investigation in wet lab experiments to assess their immunogenicity, safety, and efficacy for practical application in livestock. Our findings offer a robust framework for developing a comprehensive, broad-spectrum vaccine, potentially revolutionizing BT control and prevention strategies in the livestock industry.

## Introduction

Bluetongue (BT) is a severe arboviral disease primarily afflicting ruminants, especially sheep and bovines. It is caused by the Bluetongue virus (BTV), a member of the *Orbivirus* genus within the Reoviridae family (1, 2). Transmission of BTV occurs rapidly through the bites of *Culicoides* midges, which are blood-feeding insects. Once the virus enters the host, dendritic cells (DCs) recognize and take-up BTV, before migrating to the draining regional lymph nodes (RLNs) (3–5). In RLNs, the virus replicates before spreading to the spleen, lungs, muscles, and pulmonary artery, leading to severe tissue damage, edema, vascular thrombosis, hemorrhage, and tissue infarction (3, 6, 7). This devastating infection results in lameness, decreased production, mortality, and significant economic loss (8).

Given its high transmission rate, severity, and economic impact, controlling BTV spread among ruminants is crucial. Vaccination is an effective measure to control the spread of BTV infection. Conventional vaccines such as live attenuated vaccines (LAVs) and inactivated vaccines (IAVs) provide some protection in sheep against BT (4, 5, 9, 10). However, the concerns associated with the LAVs such as reversion to virulence, fetal malformations, and poor immune response in the case of IAVs limit their use in vaccination (7). They also continue to circulate, contributing to genetic diversity and potential reassortment with wild-type strains. Furthermore, these conventional vaccines are derived from a particular serotype, which confers serotype specific protection (4–6, 9–13). On the other hand, the virus has evolved into more than 28 different BTV serotypes, rendering conventional serotype-specific vaccines less effective (14). IAVs, while safer than LAVs, typically confer serotype-specific and often weaker immunity (7). While DNA plasmid-vector vaccines have demonstrated efficacy in laboratory mouse models (15, 16), their translation to field use in animals has been hindered by challenges such as the risk of affecting genes that control cell growth, the need for repeated doses, induction of lower immunogenicity, and the high cost of production at an industrial scale. Therefore, conventional vaccines fall short in providing comprehensive protection against different BTV serotypes, underscoring the compelling need to develop a cost-effective, broad-spectrum vaccine capable of protecting against multiple BTV serotypes.

Several pioneering studies emphasize the significance of T cell-mediated cross-reactivity due to the presence of conserved T cell epitopes in various BTV proteins (17, 18). For instance, viral vectors expressing NS1 protein have been shown to induce cross-reactive CD8+ T cell-mediated protection against multiple BTV serotypes (19–21). Additionally, studies indicate that viral vectors expressing other BTV proteins, such as NS2, VP2, and VP7, can also provide CD8+ T cell-mediated cross-protection against multiple BTV serotypes (22–24). Furthermore, direct evaluations of the relevance of using epitopes from BTV proteins in vaccine formulation have been conducted. Studies by Roja et al. (25–27) suggest that epitopes from NS1, VP2 and VP7 proteins of BTV can induce cross-reactive CD4+ and CD8+ T cell responses against multiple BTV serotypes. Similarly, many other studies have demonstrated the importance of CD4+ and CD8+ T cells that recognize epitopes on structural (VP2 and VP7) and non-structural (NS1) BTV proteins in conferring protection against BT (23, 24, 28–30). In light of these findings, vaccination designed to elicit cross-reactive T cell responses holds the potential to protect animals against several BTV serotypes.

Multi-epitope subunit vaccines represent an effective means to induce cross-reactive cell-mediated immune responses and have shown effectiveness in infectious disease and cancer control (31, 32). They are produced via recombinant DNA technology and offer robust immunogenicity, safety, and scalability. Hence, in the present study, after rigorously screening for high antigenicity, non-allergenicity, non-toxicity, and the ability to induce IFN-gamma (IFN-γ), we identified and incorporated safe and immunogenic conserved T cell epitopes into an *in silico*-based multi-epitope BTV vaccine. In our strategy, we emphasize T cell epitopes over B cell epitopes due to their documented ability to induce cross-reactive T cell-mediated immune responses against diverse virus serotypes (33–35). This phenomenon is also observed in BTV infections and vaccination of sheep (5, 29, 36–38), suggesting shared determinants for cell-mediated immunity among BTV serotypes. Notably, BTV infections elicit serotype-specific neutralizing antibodies primarily against the outer capsid, which exhibits significant variability across serotypes (29, 39), prompting the exclusion of B cell epitopes in our broad-spectrum vaccine design. Furthermore, we selected the non-structural proteins of BTV as promising vaccine targets because they are highly conserved and are also the dominant source for T cell epitopes, particularly NS1 protein (1–3, 8, 10, 11, 13, 15–19, 21, 22, 24, 27, 30, 33–36, 38–87) (22, 29). Additionally, we performed protein-protein docking and molecular dynamics simulations (MDS) to study the molecular interaction patterns and investigate the vaccine’s capacity to trigger an immune response. Therefore, our extensive investigation provides a proof of concept for future research endeavors aimed at developing effective pan-BTV vaccines, capable of conferring cross-protection against all existing BTV serotypes with the potential to prevent the spread of these virulent serotypes.

## Methodology

### Sequence retrieval and multiple sequence alignment

The full-length amino acid sequences of BTV NS proteins (NS1, NS2, and NS3) were downloaded from the available genomes of 24 serotypes of BTV at the taxonomy browser of NCBI (https://www.ncbi.nlm.nih.gov/Taxonomy/Browser/wwwtax.cgi?id=40051) by using “Bluetongue” as a search keyword. The ClustalW tool (63) embedded in MEGA X (88) was employed to perform the multiple sequence alignment (MSA) to identify the conserved CD8+ and CD4+ T cell epitopes. The output of alignment files was saved in MEGA format with an extension “.meg” or “.mega”. The amino acid sequence conservation was determined in the MEGA format files of NS1, NS2, and NS3 proteins. The amino acid sequences of these proteins were subjected to BLASTp search against the mouse and bovine proteomes to identify the amino acid similarity of BTV proteins with the host proteome.

### Murine MHC class I- and II-restricted epitope prediction

**Figure 1:**
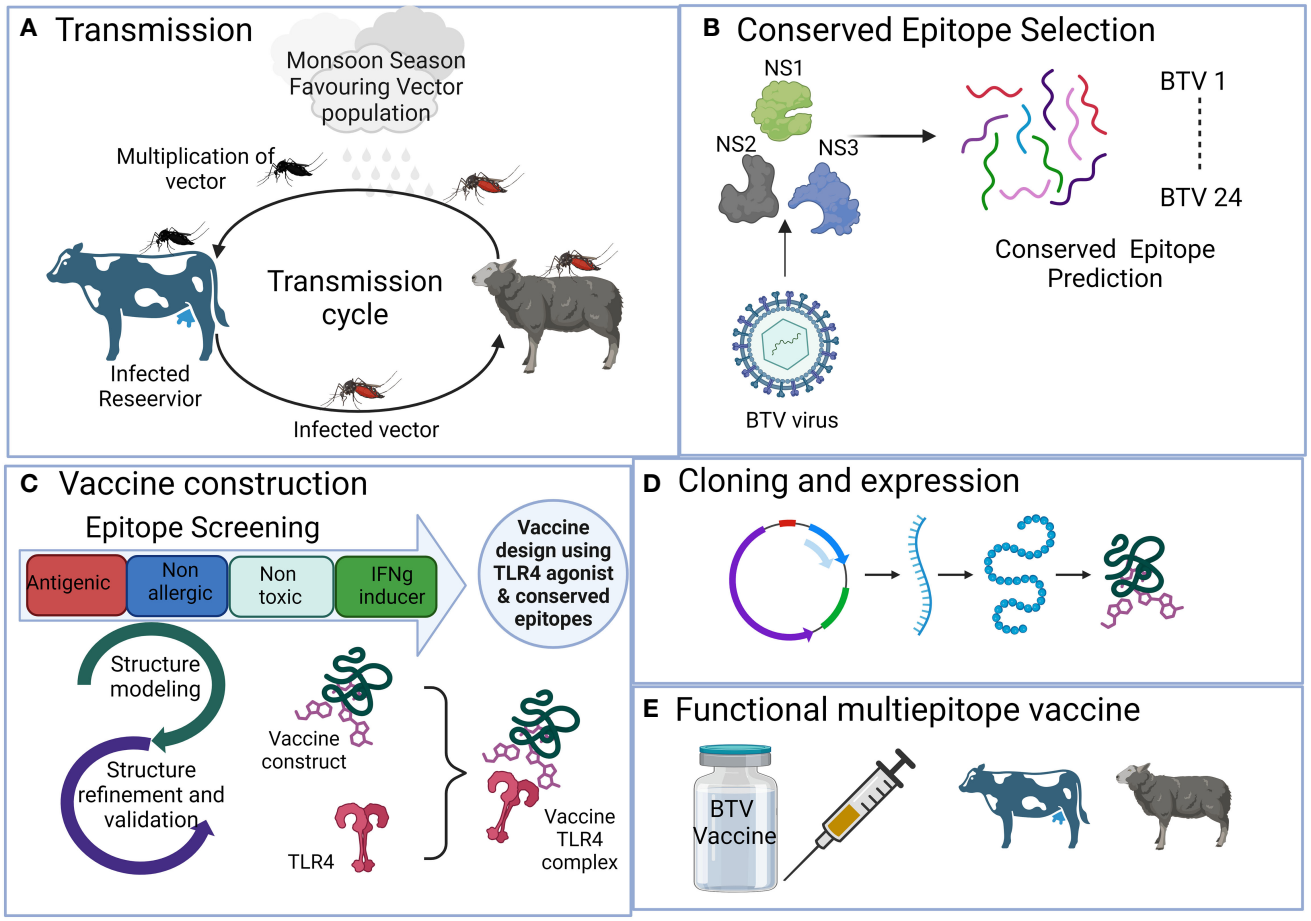
The schematic representation illustrates the process of developing a multi-valent broad-spectrum epitope vaccine against conventional 24 BTV serotypes with the aid of immunoinformatics methods. It outlines the sequential steps involved in the process, which include **(A)** Viral disease transmission and spreading from reservoir host bovines to natural host, sheep, via *Culicoides* vector. **(B)** Selection of antigenic non-structural viral proteins and prediction of conserved epitopes. **(C)** Rigorous epitope screening and vaccine construction incorporating the prediction of potent but safe antigenic epitopes. Subsequently, 3D modeling and comprehensive assessment of various construct parameters are performed, including structure modeling, molecular docking, and simulation with bovine Toll-like receptor 4 to assess affinity and interaction with the host. **(D)** Determining *in silico* cloning compatibility with prokaryotic expression systems for large-scale vaccine production. **(E)** The final outcome is the development of a broad-spectrum multi-epitope BTV vaccine that is efficient, safe, and broadly protective against all the BTV serotypes.

The immunogenicity of an antigen is significantly influenced by the affinity of interaction between T cell receptors (TCRs), epitopes, and MHC complex, particularly for viral-specific CD8+ and CD4+ T cells (12, 13). Predictions for MHC class I alleles (H2-K^b^ and H2-D^b^) in C57BL/6 mice were conducted using the “NetMHCpan BA 4.1” module of the Immune Epitope Database Epitope Analysis Resource (IEDB-AR) (http://tools.iedb.org/mhci/). The predicted MHC-I-binding epitopes were identified within the NS1, NS2, and NS3 proteins of the BTV1 serotype genome (**Figure 1**). Selection of epitopes was based on Half-maximal Inhibitory Concentration (IC50) values, with a threshold of <500 nM considered for CD8+ T cell epitopes (79) (12, 13).

Similarly, predictions for CD4+ T cell epitopes were performed for the MHC class II H2-IA^b^ allele in C57BL/6 mice, utilizing the “MHCIIpan 4.0 BA” module of the IEDB-AR tool (http://tools.iedb.org/mhcii/). IC50 values were employed as threshold parameters, with <1000 nM considered for CD4+ T cell epitopes, determining the interaction between the epitope peptide and the MHC allele (89, 90). The predictions focused on the NS1, NS2, and NS3 proteins of the BTV1 serotype genome.

### Bovine leukocyte antigen class I- and II-restricted epitope prediction

In the bovine system, CD8+ T cell epitopes within the NS1, NS2, and NS3 proteins of the BTV1 serotype were predicted for BoLA class I alleles (**Figure 1**). The prediction was conducted using the IEDB-AR server (http://tools.iedb.org/mhci/) (45), with the host species set as “cow.” For BoLA class I allele-specific CD8+ T cell epitopes, the most frequent alleles, including BoLA-1*02301, BoLA-2*01201, BoLA-3*00201, BoLA-4*02401, BoLA-6*01301 and BoLA-6*01302, were considered based on previous studies (80, 91). These alleles have been previously used for the prediction of BoLA-restricted class I epitopes for foot-and-mouth virus, whose infection resembles BT in the initial stages of infection (92). A threshold of IC50 value <500 nM was used for epitope prediction.

For BoLA class II-restricted CD4+ T cell epitope prediction, the NetBoLAIIPan 1.0 tool (https://services.healthtech.dtu.dk/service.php? NetBoLAIIpan-1.0) was employed with default settings (52). This tool generates the possible predicted T cell epitopes in 15 amino acid length with different % Rank EL scores. A threshold parameter of % Rank EL scores less than 1.0 was applied for the prediction of CD4+ T cell epitopes (52). All well-characterized class II alleles of BoLA, including BoLA-BoLA-DRB3_0101, BoLA-DRB3_1001, BoLADRB3_1101, BoLA-DRB3_1201, BoLA-DRB3_1501, BoLADRB3_1601 and BoLADRB3_2002, were selected for predicting CD4+ T cell epitopes.

### Epitope conservation

All predicted T cell epitopes, including both CD8+ and CD4+ epitopes, underwent verification for their conservation across all BTV serotypes. To accomplish this, we scrutinized the predicted epitopes located within the NS1, NS2, and NS3 proteins of BTV1 by cross-referencing them with the amino acid alignment files previously generated using MEGA-X software. Our analysis focused on ascertaining the presence and degree of conservation of these BTV1 epitopes within the alignment files encompassing NS1, NS2, and NS3 proteins across all 24 serotypes. The results of this conservation assessment are presented and discussed comprehensively within this study.

### Vaccine design

Highly conserved CD8+ and CD4+ T cell epitopes were used for the design of a multi-epitope broad-spectrum BTV vaccine (**Figure 1**). Firstly, the T cell epitopes were screened for the antigenicity, allergenicity, toxicity and IFN-γ-inducing abilities with the aid of VaxiJen v2.0 (http://www.ddg-pharmfac.net/vaxijen/VaxiJen/VaxiJen.html) (48), AllerTOP v2.0 (https://www.ddg-pharmfac.net/AllerTOP/) (47), ToxinPred (http://crdd.osdd.net/raghava/toxinpred/) (57), and IFNepitope predict (https://webs.iiitd.edu.in/raghava/ifnepitope) (46) web servers, respectively. Finally, the antigenic, non-allergic, non-toxic, and IFN-γ-inducing conserved T cell epitopes were considered for vaccine design.

The selection of linkers in our designed vaccine is based on their critical role in maintaining the structural integrity of epitopes during the vaccine’s antigen processing and presentation. Given that our vaccine is a novel protein with altered antigen presentation characteristics compared to the native protein, the chosen linkers serve to ensure efficient confirmation, cleavage, and presentation of T cell epitopes by MHC molecules on antigen-presenting cells. The linkers utilized in our design are standard choices widely employed by researchers for developing multi-epitope vaccines (67, 71, 93). Moreover, the vaccine constructs using these linkers are also validated experimentally (50). The CD8+ and CD4+ T cell epitopes were joined together with the help of ‘AAY’ and ‘GPGPG’ linkers (66).

For the enhancement of our vaccine constructs, an adjuvant sequence was strategically appended to the vaccine sequence’s N-terminal end, facilitated by the ‘EAAAK’ linker (**Figure 1**) (94). The first adjuvant utilized in our vaccine design is beta-defensin 2, recognized as a robust immunomodulator and extensively studied as a promising adjuvant molecule for Toll-like receptor 4 (TLR4) activation (65, 72, 77, 95–98). Multiple studies support its efficacy in stimulating immune responses, emphasizing its potential role in enhancing vaccine effectiveness. The second adjuvant integrated into our vaccine design is the sequence derived from the 50S ribosomal subunit (74). This sequence is strategically chosen for its ability to activate TLR4, initiating a cascade of proinflammatory cytokine production. Best vaccine construct was further determined based on the binding affinity between the vaccine molecule and the TLR4 during docking studies.

### Antigenicity, allergenicity, solubility, and physicochemical properties of the vaccine constructs

We designed a total of 4 vaccine constructs, which included 2 mouse (each with β-defensin 2 agonist or 50S ribosomal subunit adjuvant) and 2 bovine vaccines (each with β-defensin 2 agonist or 50S ribosomal subunit adjuvant). These vaccines were evaluated for their antigenicity and allergenicity as above. The solubility and physicochemical properties, including molecular weight, theoretical isoelectric point (pI), instability index (II), aliphatic index (AI), and grand average of hydropathicity index (GRAVY) of the vaccine were predicted using Protein-Sol (http://protein-sol.manchester.ac.uk/) (58) and ProtParam tool (https://web.expasy.org/protparam/), respectively.

### Immune simulation studies

We further evaluated the cytokine response by all four vaccine constructs *in silico* using the C-IMMSIM server (https://kraken.iac.rm.cnr.it/C-IMMSIM/index.php?page=1). The immune simulations were performed with the default settings with few modifications. The total number of simulation steps were set as 1050 and injections were administered at 1, 84, and 168 steps as described previously (62, 99).

### Structure modeling and evaluation

The popular Robetta server (https://robetta.bakerlab.org/) (69) was utilized to predict the three-dimensional structure of the designed vaccine constructs (**Figure 1**). The top structural models of all four vaccine constructs were further refined for protein-protein docking studies. Subsequently, we refined the modeled 3D structures using the automated GalaxyWEB server (70). The stereochemical properties of the modeled structures were assessed by analyzing phi (ϕ) and psi (Ψ) torsion angles of the Ramachandran plot using the PROCHECK program (85) embedded in protein structure validation software suite (PSVS) (https://montelionelab.chem.rpi.edu/PSVS/PSVS/).

### Preparation of the receptors and molecular docking

The ability of the receptor TLR4 to interact with vaccine constructs was analyzed by protein-protein docking (**Figure 1**). The TLR4 is well known to elicit antiviral activity (100). The 3D coordinates of mouse TLR4 (PDB ID: 4G8A) protein was obtained from the RCSB-PDB (101) in the pdb format. The bovine TLR4 (Q9GL65) was obtained from the AlphaFold protein structure database (https://alphafold.ebi.ac.uk/). Before docking, these receptor molecules were prepared with the help of MGLTools (78) and the UCSF Chimera package (82). For molecular docking studies, the ClusPro server 2.0 (102) was utilized. In the docking process, the TLR4 protein served as a receptor (chain A), while the designed vaccine constructs were used as ligand molecules (chain B). The top-ranked docking complexes were selected and the binding affinity between the receptor and ligand in the docked complexes was determined with the automated PRODIGY server (75). The PDBsum server (73) was utilized to generate the 2D interaction graphs.

### Molecular dynamics simulations

An MDS framework was established for all top four docking complexes to investigate their stability at the atomic level (**Figure 1**). The same protocol was applied to every complex, utilizing state-of-the-art MDS techniques with the AMBER99SB-ILDN protein and AMBER94 nucleic force fields (76) embedded into the GROMACS 2023 package (103) installed on a high-performance computing (HPC) infrastructure. The docking complexes were solvated using the transferable intermolecular potential 3P (TIP3P) water model. To achieve neutrality, we employed LINear Constraint Solver (LINCS) constraint algorithms for energy minimization in all systems (59). Next, the docked complexes underwent equilibration using the NVT ensemble at 300K and the NPT ensemble with the Parinello-Rahman barostat (81) coupling ensembles. Subsequently, a 100 ns MDS was conducted for all four docked complexes. 100 ns is a standard duration widely used in MD simulations of docked complexes in the context of multi-epitope vaccine development (60, 64, 104, 105). After the MDS, we analyzed the trajectories and generated plots using various modules available in the GROMACS package.

### Codon optimization and *in silico* cloning

All four designed vaccines were reverse translated to the corresponding nucleotide sequences and cloned into the pET vector using the VectorBuilder site (https://en.vectorbuilder.com/design.html) (**Figure 1**). This would be required for the evaluation of expression of recombinant proteins in *E. coli* and further functional studies such as immunogenicity, safety and studying the cross protection in laboratory settings. Firstly, we converted the amino acid sequences of the designed vaccines to nucleotide sequences through codon optimization. Codon optimization was performed using the JCat (https://www.jcat.de/) (56) and the GC content of the optimized sequences were determined using standard molecular tools online. The optimized codon sequences were virtually cloned into the pET vector.

## Results

### Differing conservation levels across non-structural proteins

**Figure 2:**
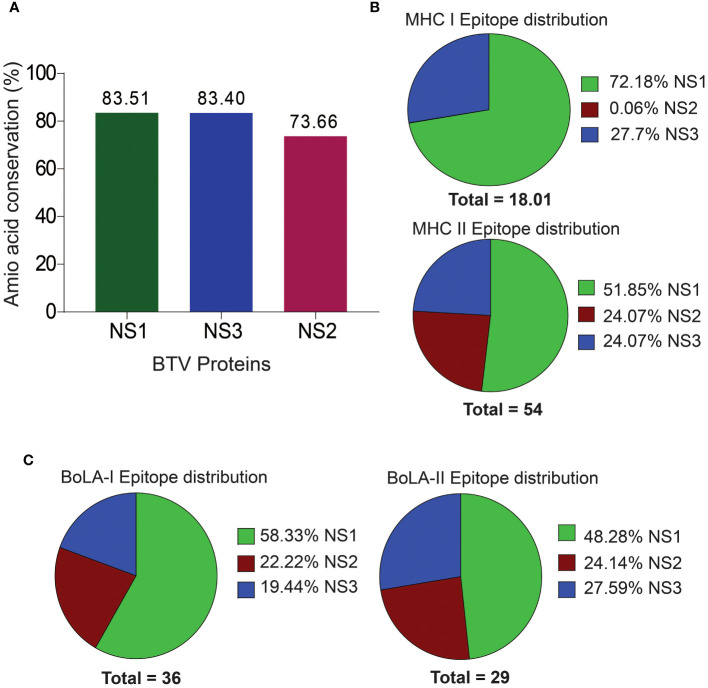
Amino Acid Sequence Conservation and T Cell Epitope Distribution in Non-Structural BTV Proteins. **(A)** Amino acid sequence conservation within the non-structural proteins, highlighting pronounced conservation in NS1, NS2, and NS3 proteins. **(B, C)**. Distribution of murine **(B)** and bovine **(C)** CD8+ and CD4+ T cell epitopes across the NS proteins.

We performed a thorough analysis of conserved amino acid sequences within the selected NS proteins of BTV, namely NS1, NS2, and NS3, employing MSA to assess the extent of conservation. The NS1, NS2, and NS3 proteins exhibited varying degrees of conservation at sequence level. Notably, NS1 emerged as the most highly conserved with an impressive amino acid sequence identity of 83.51%, followed by NS3 with 83.40% and NS2 with 73.66% sequence identities (**Figure 2A**). The outcome of MS indicates that these proteins might harbor a higher abundance of conserved T cell epitopes.

### Frequencies of T cell epitopes recognized by murine and bovine systems

Mouse models are increasingly becoming valuable for testing T cell-mediated immunity of vaccines against BT (17, 38, 106). Particularly, the C57BL/6 background mouse model has been well established for studying the pathogenesis and vaccine development for BTV (4, 5, 7, 83). Therefore, in the present study, we used the C57BL/6 MHC class I H2-D^b^ and H2-K^b^, and class II H2-IA^b^ haplotypes for predicting CD8+ and CD4+ T cell epitopes in NS1, NS2 and NS3 proteins of BTV. We obtained a total of 18 CD8+ T cell epitopes for the mouse system, which includes 13 (72.18%) and 5 (27.76%) CD8+ T cell epitopes in the NS1 and NS3 proteins [**Figure 2B**; **Supplementary Table 1 (S1)**]. Surprisingly, CD8+ T cell epitopes were not observed in the NS2 protein. Similarly, a total of 28 (51.85%), 13 (24.07%), and 13 (24.07%) CD4+ T cell epitopes (a total of 54) were identified in the NS1, NS2 and NS3 proteins, respectively (**Figure 2B**; **Supplementary Table S2**).

Like BTV’s natural host sheep (ovine), bovines, such as cattle, are also significant hosts and are closely related to ovine in genome, anatomy, immunology, and physiology. Since tools are unavailable for predicting T cell epitopes for ovine MHC alleles, we used only BoLA class I and II alleles to predict bovine epitopes. Our prediction analysis revealed a total of 36 CD8+ T cell epitopes across the NS1, NS2, and NS3 proteins of the BTV-1 serotype. These epitopes were distributed among the six BoLA class I alleles, with 21 (58.33%), 8 (22.22%), and 7 (19.44%) epitopes identified for NS1, NS2, and NS3 proteins, respectively (**Figure 2C**; **Supplementary Table S3**). For CD4+ T cell epitopes, a total of 29 epitopes were predicted, encompassing all seven BoLA class II alleles. The distribution across NS1, NS2, and NS3 proteins was observed as 14 (48.28%), 7 (24.14%), and 8 (27.59%) epitopes, respectively (**Figure 2C**; **Supplementary Table S4**).

### Conserved T cell epitope presentation by a laboratory murine model

**Figure 3:**
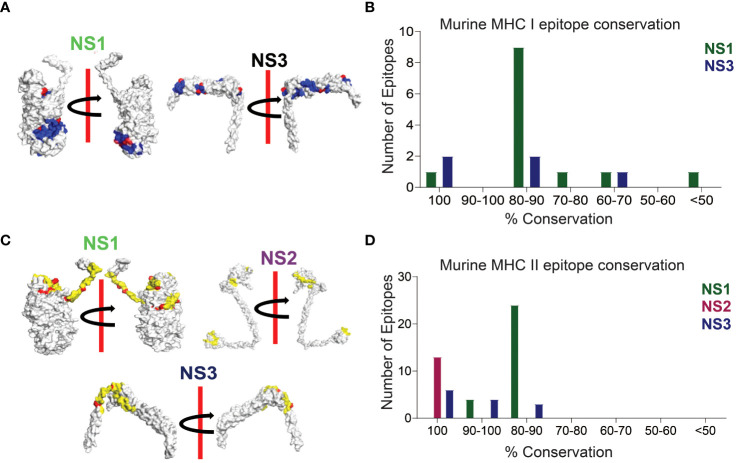
Conserved CD8+ and CD4+ T Cell Epitope Presentation in a Laboratory Murine Model. **(A)** Protein structures depicting MHC class I epitope conservation (highlighted in blue) and variations in amino acid residues within the epitopes (highlighted in red). *In silico* protein structure predictions were generated using the Robetta server, with both front and back views displayed with a 180-degree rotation. **(B)** Bar chart illustrating the extent of amino acid conservation among MHC class I-restricted CD8+ T cell epitopes within NS1 and NS3. **(C)** Protein structures showcasing MHC class II epitope conservation (highlighted in yellow) and variations in amino acid residues within the epitopes (highlighted in red). The protein structures were generated *in silico* through the Robetta server, and both front and back views are presented with a 180-degree rotation. **(D)** Bar chart presenting the degree of amino acid conservation among MHC class II-restricted CD4+ T cell epitopes within NS1, NS2, and NS3.

In our sequence alignment analysis across all 24 BTV serotypes (**Figure 3**), we identified highly conserved T cell epitopes within NS1, NS2, and NS3 proteins. Notably, the CD8+ T cell epitope H2-Kb-KHFNRYASM exhibited an exceptional 100% conservation in NS1, while two epitopes (AAFASYAEA and KAMSNTTGA) in NS3 demonstrated a similarly high level of conservation (**Figures 3A, B**; **Supplementary Table S1**). Although not all epitopes in these proteins reached 100% conservation, a substantial majority featured at least 60% conservation—a critical cutoff for considering epitopes as potentially cross-reactive (54, 107). For NS1, the amino acid conservation in the CD8+ T cell epitopes reached 88.88% (9), 77.77% (1), and 66.66% (1) (**Supplementary Table S1**). Similarly, the NS3 protein displayed two CD8+ T cell epitopes, each with 88.88% and 66.66% conserved amino acids.

In our CD4+ T cell epitope analysis, NS1 stood out with its remarkable ~83.5% sequence identity, containing 4 epitopes at 93.33% sequence identity, 18 epitopes at 86.66% sequence identity, and an additional 6 epitopes with approximately 80% sequence identity (**Figures 3C, D**; **Supplementary Table S2**). Notably, all CD4+ T cell epitopes in the NS2 protein were found to be conserved with 100% sequence identity (**Supplementary Table S2**). In the NS3 protein, 6 out of 13 CD4+ T cell epitopes exhibited 100% conservation, while the remaining 7 had sequence identities of 93.33% (4), 86.66% (2), and 80% (1) (**Supplementary Table S2**).

### Distribution of conserved CD8+ and CD4+ T cell epitopes in bovines

**Figure 4:**
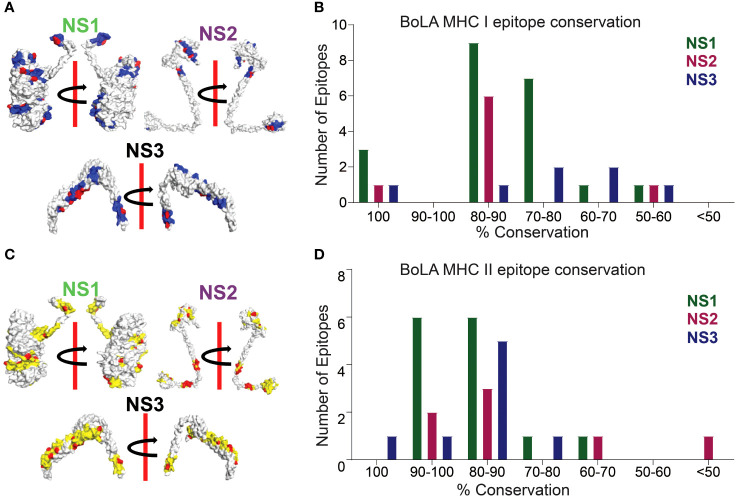
Conserved CD8+ and CD4+ T Cell Epitope Presentation by Bovine Hosts. **(A)** Protein structures illustrating MHC class I epitope conservation (highlighted in blue) and the variations in amino acid residues within the epitopes (highlighted in red). *In silico* protein structure predictions were generated using the Robetta server, with both front and back views displayed along with a 180-degree rotation. **(B)** Bar chart presenting the extent of amino acid conservation among MHC class I-restricted CD8+ T cell epitopes within NS1, NS2, and NS3. **(C)** Protein structures demonstrating MHC class II epitope conservation (highlighted in yellow) and variations in amino acid residues within the epitopes (highlighted in red). The protein structures were produced *in silico* through the Robetta server, and both front and back views are presented with a 180-degree rotation. **(D)** Bar chart depicting the degree of amino acid conservation among MHC class II-restricted CD4+ T cell epitopes within NS1, NS2, and NS3.

Similarly, in our investigation of BoLA MHC-restricted conserved T cell epitopes, we detected a discernible trend of CD8+ T cell epitope conservation within the NS1, NS2, and NS3 proteins (**Figures 4A, B**). The NS1 protein contains the highest number of 100% conserved CD8+ T cell epitopes, AMYDRETVW, KHFNRYASM and RKYNISGDY. Only one CD8+ T cell epitope is least conserved with 55.55%. Among the remaining 17 CD8+ T cell epitopes, 9 are with 88.88%, 7 with 77.77% and 1 is with 66.66% sequence identity (**Figure 4A**; **Supplementary Table S3**). In NS2, only 1 CD8+ T cell epitope is 100% conserved, while 6 had 88.88% amino acid conservation. In the NS3 protein, 1 CD8+ T cell epitope is highly conserved with 100% conservation and the remaining CD8+ T cell epitopes contain > 65% of the amino acid conservation – 1 with 88.88%, 2 with 77.77%, and 2 with 66.66% sequence identity. Notably, NS3 also harbored a 100% conserved CD4+ T cell epitope, LRQIKRHVNEQILPK.

Much like the conservation observed in CD8+ T cell epitopes, a similar pattern emerged in CD4+ T cell epitopes within the NS1, NS2, and NS3 proteins (**Figures 4C, D**). Among these, NS3 exhibited the highest conservation of CD4+ T cell epitopes, reaching 100%, followed by NS1 and NS2 (**Figure 4D**; **Supplementary Table S4**). Within NS1, 12 out of 14 predicted CD4+ T cell epitopes were conserved, with sequence identities of 93.33% and 86.66% and the remaining 2 epitopes have sequence identities of 73.33% and 66.66% (**Figure 4C**; **Supplementary Table S4**). Similarly, in NS2, all CD4+ T cell epitopes demonstrated conservation, with over 60% sequence identity. This included 93.33% for 2 epitopes, 86.66% for 1, 80% for 2, and 60% for 1, with just one epitope showing a lower conservation at 26.66% (**Figure 4C**; **Supplementary Table S4**). Notably, NS3, being highly conserved, harbored CD4+ T cell epitopes with more than 73% sequence conservation, with an identity of 100% for 1 epitope, 93.33% for 1, 86.66% for 2, 80% for 3, and 73.33% for 1 (**Figure 4C**; **Supplementary Table S4**).

### Non-structural proteins are hotspots for conserved epitopes in both murine & bovine systems

**Figure 5:**
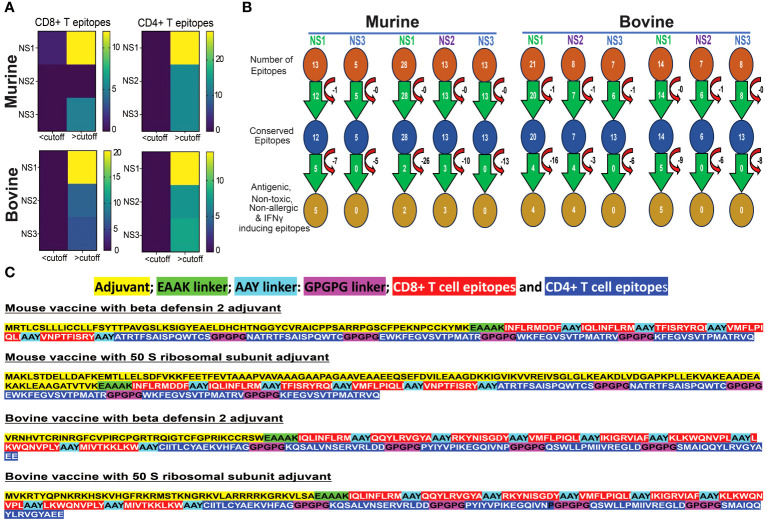
Conserved Epitopes and Multi-Epitope Broad-spectrum Vaccine Design for Bluetongue Virus. **(A)** Non-Structural Proteins Serve as Hotspots for Conserved Epitopes in both Murine and Bovine Systems. Heat maps demonstrate the increased prevalence of CD4+ and CD8+ T cell epitopes, particularly within the NS1 protein. **(B)** Schematic representation of the screening process for identifying highly conserved immunodominant epitopes, used in the development of a multi-epitope pan-BTV vaccine. **(C)** Design of the multi-epitope pan-BTV vaccine for both murine and bovine systems. The adjuvant is attached at the N-terminal of the vaccine sequence using an EAAK linker, while CD8+ and CD4+ T cell epitopes are connected with AAY and GPGPG linkers, respectively.

**Figure 5A** illustrates that NS proteins stand out as a prominent source for conserved epitopes in both murine and bovine systems. A significant number of CD8+ and CD4+ T cell epitopes, with a minimum amino acid conservation of over 60%, are predominantly located within NS1, NS2, and NS3 proteins. Notably, in the murine system, no corresponding epitopes were found in the NS2 protein for MHC class I alleles. These findings underscore the significance of NS proteins as valuable candidates for inclusion in considerations for the development of BT vaccines.

### Screening of conserved epitopes and *in silico* broad-spectrum vaccine formulation

In our screening for antigenicity, allergenicity, toxicity, and IFN-γ-inducing abilities of conserved epitopes, we identified 5 MHC class I-specific CD8+ T cell epitopes in NS1, none in NS2, and none in NS3 proteins for murine vaccine development. For MHC class II-specific CD4+ T cell epitopes, we found 2 in NS1, 3 in NS2, and none in NS3 (**Figure 5B**). For bovine vaccine development, we discovered 4 BoLA class I-specific CD8+ T cell epitopes in NS1, 4 in NS2, and none in NS3. Additionally, we found 5 BoLA class II-specific CD4+ T cell epitopes in NS1, none in NS2, and none in NS3 proteins. These filtered epitopes, exhibiting a robust anti-viral T cell response (antigenic and IFNγ-inducing) without causing any toxicity, autoreactivity, or allergenicity *in vivo*, were utilized for designing *in silico* multi-epitope broad-spectrum BTV vaccine formulations, as outlined in the Methods section (**Figure 5C**). These constructs consisted of the conserved T cell epitopes for each species linked together with suitable linkers, and either β-defensin 2 or 50S ribosomal (50 SR) subunit adjuvants added to the N-terminus region of the CTL epitopes to enhance immunogenicity (84) (**Figure 5C**). This resulted in the design of two candidate BTV vaccine constructs for mouse (mVaccine-β-def and mVaccine-50SR) and two for bovine (bVaccine-β-def and bVaccine-50SR).

### Antigenicity, allergenicity, solubility, and physicochemical properties of the vaccine constructs

The assessment of antigenicity revealed that all four vaccine constructs are deemed probable antigens. Similarly, both allergenicity and solubility analyses affirmed that these constructs are non-allergenic, ensuring their safety for vaccine development, and possess favorable solubility (**Table 1**).

**Table 1:** Immunological and physicochemical properties of the designed vaccine constructs.

Vaccine	Antigenicity	Allergenicity	Molecular weight	Isoelectric point	Instability index	Aliphatic index	GRAVY
**mVaccine-β-def**	Antigenic (**0.6964)**	Non-allergen	25204.32	9.269	27.02	69.35	0.063
**mVaccine-50 SR**	Antigenic (**0.5900)**	Non-allergen	30824.54	5.53	20.52	83.59	0.11
**bVaccine-β-def**	Antigenic (**0.6362)**	Non-allergen	26327.01	9.70	28.29	93.47	0.032
**bVaccine-50 SR**	Antigenic (**0.6098)**	Non-allergen	27111.99	10.21	37.42	90.33	-0.208

The physicochemical properties evaluation showed that mouse-vaccine with beta defensin 2 adjuvant, -50 SR subunit adjuvant, bovine-vaccine with beta defensin, and -50 SR subunit adjuvant have the molecular weights, 25204.32, 30824.54, 26327.01 and 27111.99, and theoretical isoelectric point (pI) values, 9.269., 5.53, 9.70 and 10.21, respectively (**Table 1**). Furthermore, these constructs have instability indices of 27.02, 20.52, 28.29, and 37.42, respectively, indicating that all the four vaccine constructs are stable. In addition, these constructs have higher aliphatic index values of 69.35, 83.59, 93.47, and 90.33, respectively, suggesting their thermostability. Finally, GRAVY scores indicate that mVaccine-β-def, mVaccine-50SR, and bVaccine-β-def constructs are polar in nature (0.063, 0.11, and 0.032, respectively) and bVaccine-50SR is non-polar in nature (-0.208) (**Table 1**).

The predicted cytokine response profile upon administering the vaccines was also evaluated. The computer-aided immune simulations of all the four vaccine constructs are shown in **Supplementary Figure 1**. It is important to note that the cytokine profile was generated corresponding to the human immune system. This was carried out because of the unavailability of sophisticated tools to study the cytokine response post vaccination in mouse and bovine systems. However, this data provides a guide to expected similar cytokine responses for all the vaccines in mouse and bovine hosts. The simulation plots show high levels of IFN-γ and IL-2 in all four vaccinated groups. Notably, the concentration of IL-2 is higher in vaccinated mice, particularly those immunized with mVaccine-50SR, in comparison to bovine subjects (**Supplementary Figure 1**). These findings collectively indicate the proficiency of our vaccines in eliciting robust proinflammatory responses, crucial for fostering potent cellular T cell responses essential for inducing cross-protection.

### Structural modeling and evaluation of the vaccine constructs

**Figure 6:**
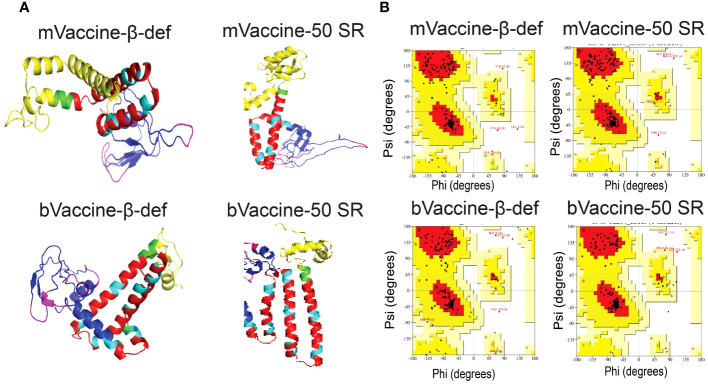
Modeling and Evaluation of Three-Dimensional Structures of the Vaccine Constructs. **(A)** Three-dimensional structures of the vaccine constructs obtained through protein modeling. **(B)** Ramachandran plots illustrating the protein models of vaccine constructs obtained through protein modeling.

In this study, we utilized the advanced tool for the structure modeling of the designed vaccine constructs (**Figure 6A**). Predicted protein models with lower RMSD values were considered for refinement. The refined protein structures improved the RMSD values of all the protein models. Structural evaluations of the 3D structure models of vaccine constructs using Ramachandran plot calculations are a popular approach and have frequently been utilized in several recent studies (108, 109). As per the general criteria of Ramachandran plot analysis, a model with ~90% of residues in the most favored regions are considered of good quality. The modeled 3D structure models were evaluated by calculating their phi (ϕ) and psi (Ψ) torsion angles using Ramachandran plot analysis. As evident from **Figure 6B**, the generated Ramachandran plots statistics for mVaccine-β-def, mVaccine-50SR, and bVaccine-β-def and bVaccine-50SR modeled structures revealed a total of 89.9, 92.7, 89.6 and 96% of residues, respectively in favorable regions, and 8, 6.1,7.8 and 3% of residues in the additionally allowed regions, respectively (**Supplementary Table 6**). Furthermore, only 0 to 1.6% of residues were found in the generously allowed regions and no single residue was found scattered in disallowed regions of the Ramachandran plots, confirming the excellent quality of the modeled 3D structures of all four vaccine constructs. The evaluation of the structural models therefore indicate that the predicted structures of the vaccine constructs are of good quality and suitable for molecular docking.

### Protein-protein docking

**Figure 7:**
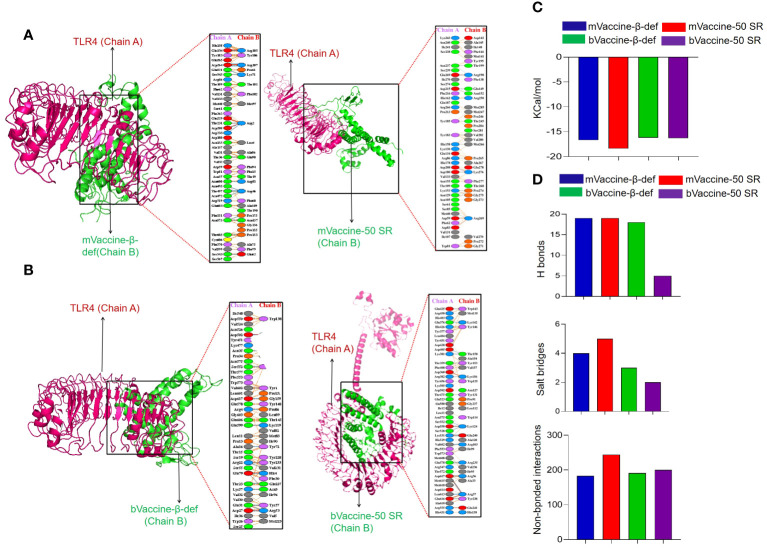
Interaction of the Vaccine Constructs with Mouse and Bovine TLRs. **(A)** Molecular interactions of the mouse vaccine constructs (chain B) with TLR4. The interaction between mouse mVaccine-β-def (left) and mVaccine-50SR (right) with TLR4 is shown in both 3-D and 2-D views. **(B)** Molecular interactions of the bovine vaccine constructs (chain B) with TLR4. The interaction between bVaccine-β-def (left) and bVaccine-50SR (right) with TLR4 is shown in both 3-D and 2-D views. **(C)** Docking scores for the murine and bovine vaccine-TLR4 docked complexes. **(D)** Bar chart illustrating the total number of salt bridges, hydrogen bonds, and non-bonded interactions between the vaccine constructs and TLR4.

Protein-protein docking has been established as one of the popular approaches for the prediction of molecular interaction patterns between the TLR molecules and vaccine constructs (110). To determine the molecular interaction between designed vaccine constructs and TLRs, we performed molecular docking. All the vaccine structures were docked against TLR4 using the ClusPro server 2.0 (**Figures 7A, B**). During the docking analysis, it was observed that the vaccines, mVaccine-β-def, mVaccine-50SR, and bVaccine-β-def and bVaccine-50SR, exhibited the Gibbs free energy (ΔG) values of –16.7, –18.4, -16.2. and –16.3 Kcal/mol, respectively (**Figure 7C**). Out of these four docked vaccine constructs, mVaccine-β-def and TLR4 docked complex ranks as the top interacting construct against its receptor protein, based on the calculated higher negative docking score. The docking complexes of these vaccine constructs with TLRs exhibit several molecular interactions including hydrogen bonds, salt bridges, and non-bonded contacts.

The 2D interaction plots were predicted using the PDBSum, revealing that the docking complex of the TLR4 and mVaccine-β-def was stabilized by 19 hydrogen bonds, 4 salt bridges, and 183 non-bonded interactions (**Figures 7A, D**). The mVaccine-50SR and TLR4 complex formed 19 hydrogen bonds, 5 salt bridges, and 244 non-bonded contacts (**Figures 7A, D**). Similarly, bVaccine-β-def and TLR4 complex formed 18 hydrogen bonds, 3 salt bridges, and 191 non-bonded contacts (**Figures 7B, D**), and bVaccine-50SR and TLR4 formed 5 hydrogen bonds, 2 salt bridges, and 200 non-bonded interactions (**Figures 7B, D**). Based on the molecular interaction patterns, the complex of bVaccine-β-def and TLR4 was found to have the second highest number of hydrogen bonds after the mVaccine-50SR-TLR4 complex. The outcome of molecular docking is consistent with the docking results of the previous studies (111–113).

### Molecular dynamics simulations on 100 ns

MDS was performed to assess the stability of the docking complexes of the vaccine and receptor complexes at atomic level on standard duration 100 ns (60, 64, 104, 105). The dynamic behavior of the simulated systems was investigated using different functions including root mean square deviation (RMSD) and root mean square fluctuation (RMSF) available in the GROMACS package.

**Figure 8:**
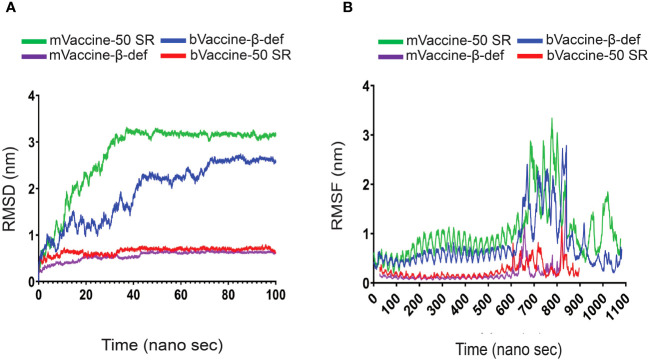
RMSD and RMSF Plots of the Docking Complexes: **(A)** Line diagram displaying the calculated RMSD complexes between the mouse and bovine vaccines and TLRs. **(B)** Line diagram illustrating the RMSF of the docking complexes between mouse and bovine vaccine constructs with their TLR4 receptors.

RMSD plot analysis is a well-established method to measure the changes in the protein structure during MDS. In the present study, calculated RMSDs of the vaccine and receptors docking complexes were graphically investigated to assess the stability at the atomic level. As evident from **Figure 8A**, vaccine and receptor docking complexes demonstrated a constant range of stability throughout the simulation on 100 ns. The average RMSD values for mVaccine-β-def, mVaccine-50SR and bVaccine-β-def and bVaccine-50SR were 0.99, 0.65, 0.34 and 1.9, respectively. As illustrated in **Figure 8A**, docking complexes of mouse vaccine constructs showed a small level of fluctuations at the starting point between 5 to 20 ns, while bovine vaccine constructs demonstrated the multiple fluctuations throughout the MD simulations on 100 ns.

The complex of bVaccine-50SR-TLR4 (red) presented as the other most fluctuated. This docking complex also showed three major fluctuations between ~5 to 20, ~25 to 40 ns, and ~55–75 ns; however, after 80 ns, it showed stability up to 100 ns on ~2.90 nm. No major fluctuations have been observed in the mVaccine-50SR-TLR4 complex. Based on the RMSD plot analysis, it can be concluded that the calculated backbone RMSDs of docking complexes indicate minimal conformational changes, and that vaccine and receptor complexes are stable at the atomic level.

The RMSF plot analysis was performed for the measurement of individual residue flexibility on a 100 ns timescale. The higher RMSF value depicts better flexibility, while the lower RMSF value indicates correct structure regions in the docking complexes of the vaccine and receptors (53). In the present study, the RMSFs of the alpha carbon atoms of all four docking complexes were studied. All four simulated complexes, mVaccine-β-def, mVaccine-50SR and bVaccine-β-def and bVaccine-50SR, exhibited a pattern of stability with several fluctuations throughout the simulation on 100 ns. The average RMSF scores of the mouse vaccine-TLR4 and mouse vaccine-TLR4 docking complexes were 1.02, 0.23, 0.18, and 0.75 respectively (**Figure 8B**). As shown in **Figure 8B**, the docking complex of both mouse and bovine vaccines with β-defensin 2 adjuvant and TLR4 docking complexes showed major fluctuations between ~600 to 839 residues. **Figure 8B** indicates that complex of bVaccine-β-def-TLR4 (blue) and mVaccine-β-def-TLR4 (purple) showed a similar region for a peak between 600 to 839 residues throughout the simulation on a 100 ns time scale; however, bVaccine-β-def-TLR4 complex (blue) demonstrated the highest peak on ~3.3 nm when compared to mVaccine-β-def-TLR4 (purple). **Figure 8B** indicates that the complex of bVaccine-50SR-TLR4 (red) and mVaccine-50SR-TLR4 (green) showed a similar region for a peak between 600 to 800 residues throughout the simulation on a 100 ns timescale; however, the complex of bVaccine-50SR-TLR4 (red) demonstrated the highest peak on ~2.8 nm compared to the mouse counterpart. Taken together, the few noted peaks observed in the vaccine-receptor complexes support the molecular docking results and suggest that all the vaccine constructs significantly interact with TLRs.

### Recombinant plasmids

**Figure 9:**
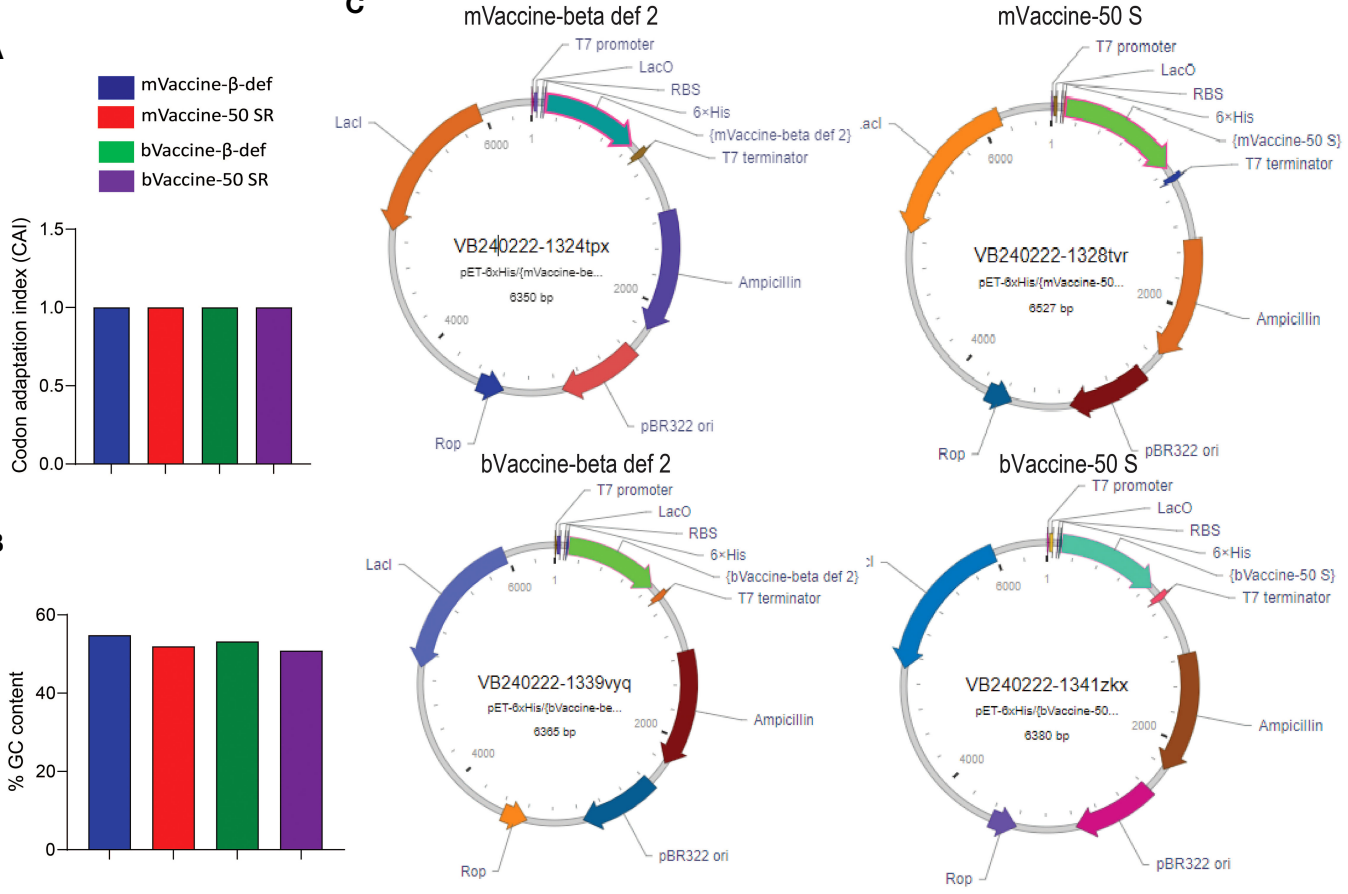
Cloning of vaccine constructs: **(A)** Codon adaptation index values of all four designed vaccine constructs. **(B)** Percent GC content in all four designed vaccine constructs. **(C)** Plasmid maps representing the cloning of the vaccine sequences into the pET vector for expression in *E*. *coli*.

After successfully validating all four vaccine candidates *in silico*, we generated the recombinant pET plasmids carrying the corresponding nucleotide sequences for protein expression and further downstream studies. Before cloning, the sequences were analyzed for their quality in terms of % GC content and codon adaptation index (CAI). Any nucleotide sequence with a %GC content of 30–70% and a CAI value of > 0.8 is considered ideal for expression in a respective host (68). Since all our constructs have a CAI of 1.0 and more than 50% GC content (**Figures 9A, B**), they are found to be in good quality for recombinant expression in *E. coli*. All four vaccine constructs were cloned under the control of the T7 promoter with a 6X His tag to aid protein purification and multi-epitope vaccine development (**Figure 9C**).

## Discussion

No prior studies have explored the application of immunoinformatics approaches for the comprehensive design of a multi-epitope vaccine capable of covering the diverse serotypes of BTV and providing cross-protection. The integration of computational tools and immunoinformatics not only facilitates the identification of these critical epitopes but also provides a strong foundation for advancing the field of pan-BTV vaccine development. The uniqueness of our research lies not only in targeting this specific pathogen but also in our extensive screening of a vast pool of T cell epitopes, aiming to maximize vaccine safety and efficacy against multiple BTV serotypes. The study utilized highly conserved epitopes meeting vaccine formulation criteria, including antigenicity, non-allergenicity, and non-toxicity. Emphasis was placed on ensuring these epitopes induce IFN-γ, known for steering immune responses towards antiviral immunity (55) (49). Incorporating both cytotoxic and T-helper cell epitopes was aimed to enhance the effectiveness of anti-BTV immunity, considering the role of T-helper cells in boosting primary and memory responses of CD8+ cytotoxic T cells (5, 7, 114).

Our final vaccine constructs exhibited highly promising attributes, encompassing antigenicity, allergenicity, solubility, and structural characteristics. Rigorous structural modeling confirmed their identity and coverage, aligning seamlessly with template structures. The Ramachandran plot analysis further emphasized the outstanding geometry of these constructs, indicative of a favorable structural conformation. The incorporation of TLR agonists, such as β-defensin 2 and the 50SR subunit, in the vaccine design play a critical role in stimulating TLR4 and triggering immune responses. TLRs, functioning as Pattern Recognition Receptors, play a pivotal role in pathogen identification, initiating both innate and adaptive immune responses. Hence, they are commonly being used in multi-epitope vaccines (65, 96, 97). Molecular docking and interaction analyses revealed substantial interactions between the vaccine constructs and TLRs, suggesting endogenous TLRs likely recognize our vaccine structures and initiate strong vaccine responses.

Vaccination stands as the most effective strategy for combating BTV infections in ruminants, reducing susceptibility and facilitating safe animal movement from enzootic regions. Cellular immunity plays a pivotal role in conferring cross-protection against different BTV serotypes (5, 6, 18, 29, 51, 83, 86, 115–117). For example, some level of heterologous protection against BTV-23 was previously achieved through BTV-1 IAVs although their impact on other serotypes has not been explored extensively (7, 90). Subsequently, similar instances of cross-protection were observed in various BTV vaccination scenarios. For instance, research indicated that vaccination against BTV-8 also conferred cross-protection against BTV-1 infection (61). Similarly, vaccination against BTV-8 was found to cross-protect against BTV-4 and BTV-1 infections (17). Moreover, vaccination targeting BTV-9, -2, and -4 was shown to cross-protect against BTV-16 infection (118). Together, these findings strongly suggest that BTV proteins encompass conserved epitopes capable of inducing cross-protection against serologically distinct BTV serotypes. Hence, our broad-spectrum vaccine approach seeks to leverage the cross-reactive protective responses observed in BTV infections and vaccinations.

Our innovative approach is poised to overcome the challenges posed by serotype-specific responses and the presence of negative regulatory proteins in previous vaccine strategies, offering a potential solution for more effective prevention and control of BTV infections. When we screened the epitopes in the non-structural proteins for the possible ideal epitopes that are antigenic, non-allergenic, non-toxic and IFN-γ inducing abilities, it was clear that not all conserved regions or epitopes in the BTV proteins are ideal for vaccine development as many conserved epitopes were allergic, toxic, or even IL-10 inducers. We found the suitable epitopes only in the NS1 and NS2 proteins (**Supplementary Table 5**; **Figure 4**). It is worth noting that all the conserved epitopes identified in NS3 are not inducers of IFN-γ but rather stimulate IL-10 responses. This finding aligns with previous experimental reports (44) that indicate the suppressive role of the NS3 protein in downregulating interferon responses. Additionally, the presence of other negative proteins like VP3 and VP4 in BTV may further impede the development of robust cross-reactive cellular responses against multiple serotypes (43). Perhaps due to the presence of these inhibitory proteins, the effectiveness of LAVs remains limited and serotype specific despite their ability to induce cellular immune response. Hence, our findings further shed light on the challenges encountered by vaccine regimens targeting specific BTV serotypes and emphasize the need for meticulous consideration in vaccine design to achieve effective prevention of BTV infections across multiple serotypes.

Notably, some of the predicted epitopes in our study have been experimentally validated by others (26, 29). For instance, the MHC class I- and II-specific CD8+ and CD4+ T-cell epitopes in NS1 demonstrated cross-reactivity among BTV4, BTV8, and BTV1 serotypes (26). While our approach successfully predicted 6 of 8 CD8+ T cell epitopes and 2 of 3 CD4+ T cell epitopes, validated as antigenic by previous researchers (26), only one CD8+ T cell epitope met our stringent criteria for epitope selection. The exclusion of other epitopes was due to concerns related to allergenicity, toxicity, or IL-10-inducing ability.

The challenges we encountered during epitope screening played a crucial role in steering our approach away from relying solely on consensus sequences from overlapping regions. We observed numerous overlapping epitope regions, particularly in CD4+ T cell epitopes. Notably, not all epitopes within these regions met our stringent criteria for vaccine design. For example, in the NS2 protein, the MHC class II-specific peptide RWEEWKFEGVSVTPMATRVQ was identified as an allergen due to the presence of a single amino acid residue (arginine) at the N-terminal end. However, three other peptides around this region (EWKFEGVSVTPMATR, WKFEGVSVTPMATRV and KFEGVSVTPMATRVQ) were found to be antigenic, non-allergenic, non-toxic, and IFN-γ inducers, making them suitable for inclusion in the vaccine design. Similarly, two BoLA specific peptides in the NS1, RAYATMFEMVRCIITLCYAEKVHFAG (non-antigenic and allergic) and FAKHFNRYASMAIQQYLRVGYAEE (antigenic and allergic), are a few examples which failed to meet the criteria of vaccine but the individual CD4+ T cell epitopes in these peptides (CIITLCYAEKVHFAG epitope in RAYATMFEMVRCIITLCYAEKVHFAG peptide region and SMAIQQYLRVGYAEE epitope in FAKHFNRYASMAIQQYLRVGYAEE peptide region) met all the requirements for the vaccine design. Nonetheless, the study by Rojas et al. (26) serves as a proof of concept, affirming that our multi-epitope vaccines have the potential to confer efficient broad-spectrum cross-protection against all BTV serotypes, given the incorporation of thoroughly screened conserved T cell epitopes in the vaccine design.

## Limitations of the study

Considering the seriousness of BT worldwide and the challenges associated with its control, this study leveraged advanced immunoinformatic tools to develop a broad-spectrum vaccine capable of preventing infection by all existing BTV serotypes. Despite the successful *in silico* development and computational evaluation of these vaccines, experimental validation is still needed. This future direction will allow us to confirm the immunogenicity and safety of these vaccines.

Another limitation of our study is the inability to develop similar vaccines for sheep, another natural host for BTV, due to the lack of T cell epitope prediction tools specific to sheep. Nevertheless, if our *in silico* vaccines are experimentally validated, it will pave the way for developing similar vaccines for sheep. For instance, a library of peptides from the NS1 and NS2 proteins could be created and evaluated for their ability to stimulate ovine BTV-NS1 and -NS2-specific T cells using high-throughput ELISpot assays. The candidate peptides could then be assessed for amino acid conservation and subjected to epitope screening to ensure they are non-allergenic, non-toxic, and IFN-γ inducers. Based on these shortlisted conserved T cell epitopes, a multi-epitope broad-spectrum BTV vaccine for sheep could ultimately be developed.

## Conclusions

This study demonstrates the feasibility of a broad-spectrum BTV vaccine by leveraging conserved T cell epitopes present in BTV proteins. Our computational analyses and *in silico* evaluations show promising outcomes for all four vaccine constructs. While acknowledging the limitations inherent to *in silico*-based vaccine studies against animal diseases, our approach signifies a substantial advancement in the quest for a broad-spectrum solution against various BTV serotypes. Current BTV vaccines face challenges such as serotype-specific limitations, safety concerns, and inadequate cross-protection. Therefore, further validation through functional assays and preclinical studies is essential to determine the immunogenicity, safety, and protective efficacy of these vaccines *in vivo*. Despite these challenges, our novel approach offers significant potential for controlling the global spread of BTV and preventing outbreaks of new serotypes, addressing the critical need for comprehensive protection against multiple BTV serotypes in ruminants.

## Data availability statement

The original contributions presented in the study are included in the article/**Supplementary Material**. Further inquiries can be directed to the corresponding author.

## Author contributions

HK: Data curation, Formal analysis, Methodology, Software, Validation, Visualization, Writing – original draft, Writing – review & editing. MD: Methodology, Software, Validation, Visualization, Writing – review & editing. AK: Methodology, Software, Validation, Visualization, Writing – review & editing. RN: Data curation, Formal analysis, Software, Validation, Visualization, Writing – review & editing. TK: Validation, Visualization, Writing – review & editing. KS: Formal analysis, Writing – review & editing. DK: Formal analysis, Writing – review & editing. PC: Formal analysis, Writing – review & editing. CU: Conceptualization, Formal analysis, Funding acquisition, Investigation, Project administration, Supervision, Visualization, Writing – review & editing.
